# Robust and Efficient CPU-Based RGB-D Scene Reconstruction

**DOI:** 10.3390/s18113652

**Published:** 2018-10-28

**Authors:** Jianwei Li, Wei Gao, Heping Li, Fulin Tang, Yihong Wu

**Affiliations:** 1 National Laboratory of Pattern Recognition, Institute of Automation, Chinese Academy of Sciences, Beijing 100190, China; jianwei.li@nlpr.ia.ac.cn (J.L.); heping.li@ia.ac.cn (H.L.); fulin.tang@nlpr.ia.ac.cn (F.T.); yhwu@nlpr.ia.ac.cn (Y.W.); 2 School of Artificial Intelligence, University of Chinese Academy of Sciences, Beijing 100049, China

**Keywords:** 3D reconstruction, camera tracking, volumetric integration, simultaneous localization and mapping (SLAM)

## Abstract

3D scene reconstruction is an important topic in computer vision. A complete scene is reconstructed from views acquired along the camera trajectory, each view containing a small part of the scene. Tracking in textureless scenes is well known to be a Gordian knot of camera tracking, and how to obtain accurate 3D models quickly is a major challenge for existing systems. For the application of robotics, we propose a robust CPU-based approach to reconstruct indoor scenes efficiently with a consumer RGB-D camera. The proposed approach bridges feature-based camera tracking and volumetric-based data integration together and has a good reconstruction performance in terms of both robustness and efficiency. The key points in our approach include: (i) a robust and fast camera tracking method combining points and edges, which improves tracking stability in textureless scenes; (ii) an efficient data fusion strategy to select camera views and integrate RGB-D images on multiple scales, which enhances the efficiency of volumetric integration; (iii) a novel RGB-D scene reconstruction system, which can be quickly implemented on a standard CPU. Experimental results demonstrate that our approach reconstructs scenes with higher robustness and efficiency compared to state-of-the-art reconstruction systems.

## 1. Introduction

3D scene reconstruction is an important topic in computer vision with many applications, such as robotics and augmented reality. The emergence of consumer RGB-D cameras, such as Microsoft Kinect, Asus Xtion and Structure Sensor, provides an opportunity to develop indoor scene reconstruction systems conveniently.

KinectFusion [1,2] is an outstanding method to generate photorealistic dense 3D models on a GPU. It uses a volumetric representation by the Truncated Signed Distance Function (TSDF) [3] to represent the scenes and in conjunction with fast Iterative Closest Point (ICP) [4] pose estimation to provide a real-time fused dense model. Although KinectFusion has many advantages such as algorithmic simplicity, it also has some disadvantages in camera tracking and volumetric representation. For camera tracking, it suffers from tracking drift accumulation, and the efficiency of ICP algorithm is computationally costly, as in each iteration, the nearest neighbors between two point clouds have to determined. For volumetric representation, TSDF is represented as a regular grid, and the memory consumption and computation time grows cubically with the resolution.

In the past few years, researchers have explored a number of online [5,6,7,8,9,10] and offline [11,12,13,14,15,16] approaches to address these issues: Kintinuous [6], ElasticFusion [9], InfiniTAMv3 [10] and BundleFusion [14] address accumulated tracking drift by detecting loop closures; Choi et al.’s method [12,13] and 3DMatch [11] reconstruct local smooth scene fragments and globally align them together with 3D features to obtain high-quality 3D reconstruction; DVOSLAM [7,8] proposes a novel direct method by minimizing the photometric error, which outperforms the dense ICP-based method in terms of efficiency.

In contrast to dense ICP-based tracking methods, sparse feature-based methods extract features in RGB images and estimate the camera motion between the images. They are more efficient and widely used in the sparse Simultaneous Localization and Mapping (SLAM) system. In this paper, we present a new CPU-based RGB-D indoor scene reconstruction framework, which combines dense volumetric integration with a sparse feature-based tracking method and can be applied to indoor scene reconstruction with high robustness and efficiency. The main contributions of our work are:

1. A fast camera tracking method combining points and edges, by which the tracking stability in textureless scenes is improved;
2. An efficient data fusion strategy based on a novel camera view selection algorithm, by which the performance of volumetric integration is enhanced.
3. A novel RGB-D scene reconstruction system, which can be quickly implemented on a standard CPU.

The rest of the paper is organized as follows: Section 2 introduces the related work and motivation of our research. Section 3 gives an overview of our scene reconstruction system. The details of the proposed method are presented in Section 4. Section 5 describes experiment results and discussions, while Section 6 presents some concluding remarks.

## 2. Related Work

Many methods are designed for robust camera tracking and efficient volumetric integration. In this section, we briefly discuss the related work and then state the detailed motivations of our approach.

### 2.1. Camera Tracking

A remarkable feature-based camera tracking method is proposed in ORB-SLAM [17,18,19], which is an accurate and efficient system and can work in real time on standard CPUs. It is prone to fail when dealing with textureless images or when feature points temporarily vanish due to motion blur. Since lines are abundant in the indoor environment and less sensitive to lighting variation than points, some systems [20,21,22,23,24,25,26] estimate the camera location by line feature or edge information.

StructSLAM [20] extends the standard visual SLAM method to adopt the building structure lines with a parametrization method that represents the Structure lines in dominant directions. Lu et al. [21] extracted 3D point and lines from RGB-D data, analyzed their measurement uncertainties and computed camera motion using maximum likelihood estimation. Zhang et al. [22] presented a graph-based visual SLAM system using straight lines as features with a stereo sensor. PL-SLAM [23,24] proposes solutions that simultaneously leverage point and line information with a monocular and a stereo sensor, respectively. Those methods are less efficient because the detection and matching for line feature are time consuming. Unlike those methods, Edge VO [25] develops a simple and efficient edge-based tracking method without any back-end optimization. To improve the accuracy, Edge SLAM [26] extends it with two-view initialization and local optimization, but reduces its efficiency.

Inspired by the above methods, we combine the advantages of edge tracking and feature-based SLAM technology and design a robust and fast camera tracking method combining points and edges to improve the stability of camera tracking.

### 2.2. Volumetric Integration

Volumetric methods provide efficient and simple ways of integrating multiple RGB-D images into a complete 3D model. The original idea of volumetric 3D reconstruction from depth images dates back to volumetric data integration [3]. Later, the advent of consumer RGB-D cameras and massively parallel processors in GPUs led to the seminal KinectFusion system and has inspired a wide range of further work. One of the major limitations of volumetric approaches is their lack of scalability due to reliance on a uniform grid, and they can therefore only handle small scenes. Exploiting the sparsity subdivision strategies has become a research focus.

Kintinuous [5] permits the area mapped by the TSDF to move over time, which allows continuously augmenting the reconstructed surface in an incremental fashion as the camera translates and rotates in the real world. Fastfusion [27,28] proposes an efficient octree data structure that allows for fast TSDF updates and incremental meshing and that runs on a standard CPU in real time. InfiniTAM [29,30,31] uses a simple spatial hashing scheme that compresses space and allows for real-time access and updates of implicit surface data, without the need for a regular or hierarchical grid data structure.

The above methods ignore the fact that volumetric integration based on TSDF is a weighted average process. If too many redundant data are fused, the computing resources are wasted, while the surface mesh may be over-smoothed or polluted by unnecessary noise. In order to further improve the performance of volumetric integration, we propose a camera view selection algorithm to prune away redundant camera views and then quickly integrate the selected RGB-D images with multi-scale TSDF.

## 3. System Overview

**Figure 1:**
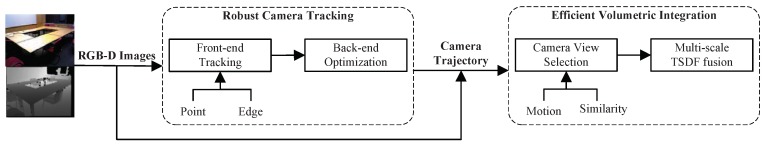
The pipeline of the proposed CPU-based 3D reconstruction system, which consists of two main stages: robust camera tracking and efficient volumetric integration. TSDF, Truncated Signed Distance Function.

A schematic overview of our approach is shown in Figure 1. The proposed system consists of two main stages: robust camera tracking and efficient volumetric integration. Each stage is briefly described as follows:

Camera tracking is to localize the camera and contains front-end tracking and back-end optimization. We perform the tracking thread with both point and edge correspondence to ensure the reliability for textureless scenes. Local mapping and loop closing are used to optimize the tracking results. The former manages the local map, and the latter detects large loops and corrects accumulated drift by pose-graph optimization.

Volumetric integration is to fuse RGB-D images of different camera views into a scene model. The output of camera tracking is a complete camera trajectory; however, it is unnecessary to use all camera views. We prune away redundant views with a novel camera view selection method based on camera motion and image similarity detection and then integrate the selected RGB-D images with adaptive multi-scale TSDF efficiently. The final mesh model is extracted with the marching cubes algorithm [32].

## 4. The Proposed Methods

The proposed methods consist of two key components, i.e., tracking via points and edges and efficient data fusion. The following subsections describe them separately.

### 4.1. Tracking via Points and Edges

The goal of camera tracking is to find the transformation T that maps the previous image into the new one. To improve the tracking reliability for textureless scenes, we implement the tracking thread with both point and edge correspondence. The transformation is estimated with two types of errors: feature point re-projection error in Equation (1) and geometrical distance error of the edge in Equation (3). Points are matched by re-projection error, which is defined as:

(1) $$\mathrm{Ep}=X^{{}^{\prime }}-KTX,$$

where X denotes the position of the 3D point; $X^{{}^{\prime }}$ denotes the position of the matched point; and K is the camera intrinsic matrix:

(2) $$\begin{array}{c} K=\left[\begin{array}{ccc} f_{u} & 0 & u_{o}\\ 0 & f_{v} & v_{o}\\ 0 & 0 & 1 \end{array}\right] \end{array}$$

Edges are matched with a warping transformation based on geometrical distance estimation [25]. The geometrical distance error is:

(3) $$\mathrm{Ee}=\left(\tau \left(K,T,x\right)-x\right)\vec{n},$$

where x is the pixel on the edge map; $\vec{n}$ is the direction of the gradient; and τ is the warping transformation [7] between consecutive frames, which is constructed as follows:

- First, 3D point p corresponding to the pixel $x={\left(u,v\right)}^{T}$ on the edge map is reconstructed using the inverse of the projection function $\pi^{-1}$ as:
  (4) $$\begin{array}{c} p=\pi^{-1}\left(K,x,z\left(x\right)\right)=z\left(x\right)\left(\frac{u+u_{o}}{f_{u}},\frac{v+v_{o}}{f_{v}},1\right), \end{array}$$
  where z(x) is the depth value of pixel x in the first depth frame.
- Second, the 3D point in the second frame is given as: T(g(ξ),p), where g(ξ) represents the transformation by the Lie algebra se(3) associated with the group SE(3). When the second camera observes the transformed point $q={\left(x_{q},y_{q},z_{q}\right)}^{T}$, we obtain the warped pixel coordinates:
  (5) $$\begin{array}{c} \pi \left(T\left(g\left(\xi \right),p\right)\right)={\left(\frac{f_{u}x_{q}}{z_{q}}-u_{o},\frac{f_{v}y_{q}}{z_{q}}-v_{o}\right)}^{T} \end{array}$$
- Finally, the full warping function is given as:
  (6) $$\begin{array}{c} \tau \left(K,\xi ,x\right)=\pi \left(T\left(g\left(\xi \right),p\right)\right)=\pi (T\left(g\left(\xi \right),\pi^{-1}\left(K,x,z\left(x\right)\right)\right)) \end{array}$$

Considering both feature point re-projection error (Ep) and geometrical distance error (Ee), we get transformation T by minimizing the cost function as:

(7) $$arg\min_{T}\lambda \sum_{i\in X_{c}}\rho_{p}(∥{\mathrm{Ep}}_{i}{∥}^{2})+\left(1-\lambda \right)\sum_{j\in X_{c}}\rho_{e}(∥{\mathrm{Ee}}_{j}{∥}^{2}),$$

where $X_{c}$ denotes the correspondence set of consecutive frames; $\rho_{p}$ and $\rho_{e}$ are the Huber function; λ∈[0,1] is the weighting coefficient. The cost function is minimized using iterations of classical Levenberg–Marquardt. The Huber function is introduced to reduce the effect of outliers. The choice of λ depends on the richness of texture features in the scene. In our experiments, we set it as:

(8) $$\lambda =\left\{\begin{array}{cc} 1, & N>N_{max}\\ \frac{N-N_{min}}{N_{max}-N_{min}}, & N_{min}\leq N\leq N_{max}\\ 0, & N<N_{min} \end{array}\right.$$

where *N* is the number of ORB features extracted per frame; $N_{min}$ and $N_{max}$ are the minimum and maximum thresholds.

**Figure 2:**
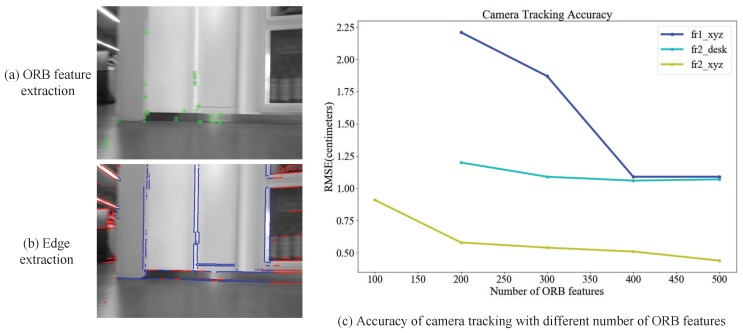
(**a**,**b**) Comparison of ORBfeature extraction and edge extraction on an RGB image. Green denotes the ORB feature points; blue denotes edges with low uncertainty; red denotes edges with high uncertainty. (**c**) Variations of the camera tracking accuracy (Absolute Trajectory (ATE) RMSE in centimeters) with different numbers of ORB features extracted per frame.

For each input RGB image, we extract points using ORB features [33] and extract edges by the DoG-based detector [34] due to its robustness in illumination and contrast changes. Figure 2a,b shows a comparison of ORB feature extraction and edge extraction on an RGB image. It can be seen that the extracted number of edges is more than the number of ORB feature points in motion blur and low texture scenes. Figure 2c shows a variation of the estimated camera trajectories with different numbers (100, 200, 300, 400 and 500) of ORB features in each frame on three sequences of the TUMRGB-D dataset [35]. The vertical axis indicates the accuracy of camera tracking using Absolute Trajectory (ATE) Root Mean Squared Error (RMSE in centimeters). When the number of ORB features in each frames is 100, the camera tracking is lost on the fr1_xyz and fr2_desk sequences. The trend of line chart indicates that the accuracy of camera tracking increases with the number of extracted ORB features and tends to be stable. Based on this experimental analysis, $N_{min}$ and $N_{max}$ are set to 200 and 400 in our experiments.

To accelerate the calculation, depth information is employed during initialization and matching processes. Due to the limitation of structure light technology, the depth value captured by the RGB-D camera such as Microsoft Kinect on the structure edges usually contains large error even when the texture is evident. We test the depth *z* from the depth image by: $z_{min}\leq z\leq z_{max}$ to select a reliable value. The choice of $z_{min}$ and $z_{max}$ depends on the parameters of the RGB-D camera. If the depth value is beyond the range, it is estimated through the standard EKF proposed in Edge VO [25]. Besides, we estimate the Standard Deviation (STD) σ of the depth noise for each pixel x based on the noise model [36]. For Microsoft Kinect, we set $z_{min}$ and $z_{max}$ to be 0.5 and four (in meters), respectively, and calculate the noise model as follows:

(9) $$\sigma =0.0012+0.0019{\left(z\left(x\right)-0.4\right)}^{2}+\frac{0.0001}{\sqrt{z(x)}}\frac{{\theta (x)}^{2}}{{\frac{\pi }{2}-\theta \left(x\right)}^{2}},$$

where z(x) is the depth value and θ(x) is the angle between the surface normal and *z* axis on pixel x. We use this noise model to analysis the uncertainty of depth values on extracted edges and eliminate edges with poor depth values.

### 4.2. Efficient Data Fusion

After camera tracking, we integrate RGB-D images with camera poses into a global model by multi-scale TSDF [27,28]. TSDF is discretized into a voxel grid to represent a physical volume of space. For a given voxel v in the fused scene model *F*, the corresponding signed distance value F(v) is computed with *r* views:

(10) $$F\left(v\right)=\frac{\sum_{i=1}^{r}f_{i}\left(v\right)w_{i}\left(v\right)}{W(v)},W\left(v\right)=\sum_{i=1}^{r}w_{i}\left(v\right).$$

Signed distance function $f_{i}\left(v\right)$ is the projective distance between a voxel and the *i*th depth frame and is defined as:

(11) $$max\{min\{\Phi ,|{\left[K^{-1}z_{i}\left(x\right){\left[x^{T},1\right]}^{T}\right]}_{z}-{\left[v\right]}_{z}|\},-\Phi \},$$

where x=π(Kv) is the pixel into which the voxel center projects and Φ is the truncation threshold. We compute the distance along the principal (Z) axis of the camera frame using the z component denoted as [.]z. Weighting function $w_{i}\left(v\right)$ represents the confidence in the accuracy of the distance, which is assigned as follows:

(12) $$w_{i}\left(v\right)=\left\{\begin{array}{cc} 1, & f_{i}\left(v\right)<\delta \\ \frac{\Phi -f_{i}\left(v\right)}{\Phi -\delta }, & \delta \leq f_{i}\left(v\right)\leq \Phi \\ 0, & f_{i}\left(v\right)>\Phi \end{array}\right.$$

where δ is one tenth of the voxel resolution.

Considering the fact that the distances from the camera to different objects in the scene are different, the geometry information should be stored at different resolution to get an accurate and efficient volumetric integration. Taking the consumer RGB-D camera Kinect used in this paper for example, the measurement error increases with the distance from points to the principal axis. In order to quickly obtain scene models with sufficient geometrical details, we use a multiple levels octree structure to store the multi-scale TSDF and update TSDF at a higher resolution for points near the camera, while a lower resolution for points far away. The geometry is stored in small cubic volumes (bricks), consisting of $8^{3}$ voxels. Each voxel stores the truncated signed distance, the weight and the color. All the bricks in the octree have the same size, while having different scales. The brick’s scale $s_{l}$ is set as: $s_{l}=exp_{2}\left\lfloor log_{2}max\left\{z_{i},1\right\}\right\rfloor$, where *l* is the level of the octree and $z_{i}$ is the depth value (in meters). The choice of Φ depends on the noise of the camera. We set it to be twice the voxel scale of the grid resolution.

As can be seen from Equation (10), TSDF fusion is a weighted average process. Even small errors of camera pose will make the TSDF model blurry and consequently lose fine details. Fusing too much redundant data has no benefit in improving the precision of the model. If too many redundant or similar data are fused repeatedly, not only the computing resources are wasted, but also the surface mesh may be polluted by unnecessary noise. Therefore, we prune away redundant camera views before volumetric integration.

The purpose of camera view selection is to remove redundant views caused by the camera’s slow motion and repeated views. Slow motion can be detected through the relative rotation and translational velocity between consecutive frames. Repeated views are determined by loop closure and con-visibility information between non-consecutive frames. The proposed camera view selection algorithm is illustrated in Algorithm 1. The complete trajectory with *n* views is reduced to a new trajectory with *r* views. As transformation $T_{i}=\left[R_{i}|t_{i}\right]$ contains camera motion information, the variables in this algorithm are calculated as follows:

- Three Euler angles $\alpha_{i}$, $\beta_{i}$ and $\gamma_{i}$ are computed by relative rotation between consecutive frames:
  (13) $$R_{i+1,i}=R_{i+1,i}^{z}\left(\alpha_{i}\right)R_{i+1,i}^{y}\left(\beta_{i}\right)R_{i+1,i}^{x}\left(\gamma_{i}\right)$$
  where $\alpha_{i}$, $\beta_{i}$ and $\gamma_{i}$ represent the yaw, pitch and roll angles, respectively;
- Translational velocity $v_{i}$ is computed by:
  (14) $$v_{i}=\left(t_{i+1}-t_{i}\right)$$
- Loop closure key frames are detected in camera tracking;
- The similarity ratio $\rho_{i,j}$ between the *i*th and *j*th frame is measured by con-visibility content information [37] and defined as:
  (15) $$\rho_{i,j}=\frac{n_{i}}{n_{j}}$$
  where $n_{i}$ and $n_{j}$ are the number of available pixels in *i*th and *j*th depth images at the *i*th frame coordinate system.

Loop closure [18] is in charge of detecting loops to reduce the cumulative errors in camera tracking. We make the key frames of loop closure as marks and use them as a basic condition to determine the repeated regions. For each depth image $D_{i}$, we only calculate the similarity ratios between the current *i*th frame and each *j*th loop closure frame in Algorithm 1. We assume that the regions with closer content have good consistency. By measuring the con-visibility information of depth images between the *i*th frame and *j*th frame, we estimate the similarity of visual contents between them and obtain a similarity ratio $\rho_{i,j}$ through Equation (15).

The selection of motion thresholds depends on the movement of the RGB-D camera. In our experiments, angle thresholds ($Th_{\alpha }$, $Th_{\beta }$ and $Th_{\gamma }$) are fixed to 0.005 (degree); velocity threshold $Th_{v}$ is set to 0.2 or 0.5 (centimeter); and similarity threshold $Th_{\rho }$ is fixed to 0.85.

**Algorithm 1** Camera view selection.**Input:** The complete trajectory $T^{n}=\left\{T_{i}\right\},i\in \left[1,n\right]$; the list of loop closure key frames $L=\left\{j\right\},j\in \left[1,l\right]$.**Output:** The reduced trajectory $T^{r}=\left\{T_{k}\right\},k\in \left[1,r\right]$.
1:**for** each input frame i∈[1,n] **do**2:    Compute $\alpha_{i}$, $\beta_{i}$, $\gamma_{i}$, $v_{i}$;3:    **if** ($|\alpha_{i}|\leq Th_{\alpha }$ or $|\beta_{i}|\leq Th_{\beta }$ or $|\gamma_{i}|\leq Th_{\gamma }$) and $\left|\right|v_{i}\left|\right|\leq Th_{v}$ **then**4:        Prune away $T_{i}$;5:    **else if** i≠j **then**6:        **for** each loop closure key frame j∈[1,l] **do**7:            Compute the con-visibility ratio $\rho_{i,j}$;8:            **if** $\rho_{i,j}\geq Th_{\rho }$ **then**9:                Prune away $T_{i}$;10:    **else**11:        Save $T_{i}$;12:**return** The reduced trajectory $T_{k}=T_{i}$;


## 5. Experiments

To illustrate the robustness and efficiency of the proposed approach, we have carried out some experiments both on synthetic and real-world scenes. The quantitative and qualitative comparisons are performed with a series of state-of-the-art systems. For all experiments, we run our system on a standard desktop PC with an Intel Core i7-4790 3.6-GHz CPU. For camera tracking, ORB-SLAM [18,19], PL-SLAM [24], Edge VO [25] and Edge SLAM [26] are run on a CPU. For 3D reconstruction, Kintinuous [6], Choi et al.’s method [12], ElasticFusion and BundleFusion are run on a GPU.

### 5.1. Camera Tracking

For camera tracking, we compare our method with several related systems (ORB-SLAM, Edge VO, Edge SLAM and PL-SLAM) in terms of tracking accuracy and computing speed on the TUM RGB-D dataset. Table 1 reports the accuracy of camera tracking (ATE RMSE in centimeters). Note that the results of ORB-SLAM (monocular), Edge VO, Edge SLAM and PL-SLAM are quoted from corresponding papers. The results show that our tracking method is superior to others in terms of accuracy and robustness on the TUM RGB-D dataset. Our method obtains good tracking accuracies and shows robustness especially on textureless scenes (fr3_snt_far and fr3_snt_near). A comparison of computing speed for camera tracking is given in Table 2. Note that the speeds of other methods are quoted from corresponding papers using the same operating environments as ours. Since edge extraction and matching are faster than line features, the mean tracking speed of our method is higher than PL-SLAM. Besides, initial pose estimation is accelerated with the help of depth information. The total tracking speed of our method can reach 58Hz on an Intel Core i7-4790 CPU when λ=1.

To further compare the robustness and accuracy of camera tracking with points and edges, experiments are also conducted on eight sequences (Living Room kt0–3 and Office kt0–3) of the ICL-NUIM Absolute Trajectory (ATE) dataset [38] and four sequences (Living Room 1–2 and Office 1–2) of the Augmented ICL-NUIM dataset [12]. Table 3 reports the accuracy of camera tracking (ATE RMSE in centimeters) with different tracking methods: tracking via points (ORB-SLAM [19]), tracking via edges (Edge VO [25]) and tracking via points and edges (our method). Note that Edge SLAM is not open source, so we cannot compare it on the ICL-NUIM dataset and the Augmented ICL-NUIM dataset. All the results in Table 3 are provided by our experiments on an Intel Core i7-4790 CPU. The results indicate that tracking via points and edges has higher robustness than tracking with points or edges, respectively.

### 5.2. Volumetric Integration

**Figure 3:**
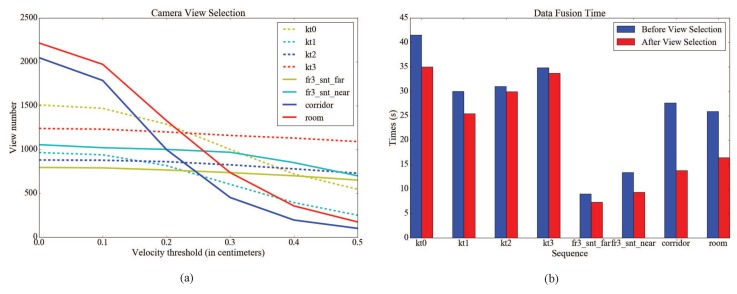
The variation of the number of camera views and data fusion time. (**a**): variation of the number of camera views before and after view selection with different velocity thresholds. (**b**): variation of data fusion time before and after view selection (ICL-NUIM and our datasets: *v* = 0.2; TUM RGB-D dataset: *v* = 0.5).

For volumetric integration, we carry out experiments to validate the camera view selection method and the efficiency of data fusion. Figure 3a shows a variation of view numbers before and after camera view selection with different velocity thresholds on ICL-NUIM living room sequences (kt0–3), the TUM RGB-D dataset (fr3_snt_f and fr3_snt_n) and our dataset (corridor and room). Fr3_snt_f and fr3_snt_n are manually scanned, while corridor and room are scanned through a robot equipped with an RGB-D camera. The effect of camera view selection on our dataset is very obvious since the sequences contain some loop closures. Figure 3b shows a comparison of data fusion time before and after camera view selection. The average time of data fusion is reduced by 21.7% on an Intel Core i7-4790 CPU. Note that the fusion speed on real-world scene (TUM RGB-D and our dataset) is faster than the synthetic scene (ICL-NUIM) because the number of valid depths is less.

**Figure 4:**
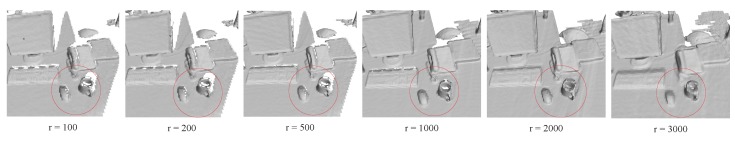
The enlarged views of reconstruction results with different numbers (100, 200, 500, 1000, 2000 and 3000) of camera views on the fr2_xyz sequence of the TUM RGB-D dataset.

To justify the reasonableness of camera view selection, we have carried out an experiment with different numbers (100, 200, 500, 1000, 2000 and 3000) of camera views on the fr2_xyz sequence of the TUM RGB-D dataset. Note that the camera pose errors are very small and the mesh models are fused by standard TSDF. The enlarged views of reconstruction results are shown in Figure 4. The models look very similar and have some missing areas due to occlusion when *r* is 100, 200 and 500. The reconstructed details are best when *r* is 1000 and get worse when *r* increases to 2000 and 3000. The results indicate that fusing too many redundant data has no benefit to improve the precision of the model.

**Figure 5:**
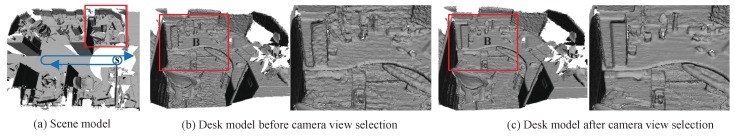
Comparison of the reconstruction results for a desk scene before and after camera view selection. Note that the mesh model is fused by standard TSDF.

Figure 5 demonstrates the reconstruction performance on a small indoor desk scene before and after camera view selection. Figure 5a shows a complete scene model, which is scanned through a robot equipped with Microsoft Kinect. The robot started at the origin *s*, moved from right to left and finally returned to the origin *s*. The trajectory and moving direction of the camera are marked with blue lines and arrows. The desk region *A* marked by the red box is a loop closure region, which has been scanned repeatedly. Figure 5b,c show the models of desk region *A* before and after camera view selection. As can be seen from the enlarged view of region *B*, the accuracy of the reconstructed surface is enhanced after camera view selection, because redundant data are removed. Note that the mesh models are fused by standard TSDF.

### 5.3. 3D Reconstruction

**Figure 6:**
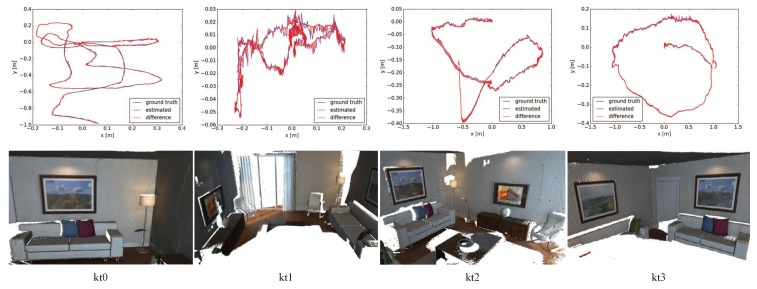
Reconstruction results with the proposed approach on ICL-NUIM living room sequences, the first row for estimated camera trajectories compared with the ground truth and the second row for surface reconstruction models.

To evaluate the proposed 3D reconstruction system quantitatively, we carry out experiments on four living room sequences (kt0–3) of the ICL-NUIM dataset. Table 4 reports the accuracy of camera trajectories (ATE RMSE in centimeters) and surface reconstruction (median distances in centimeters). Note that the results of Kintinuous, Choi et al.’s method [12], ElasticFusion, InfiniTAM v3, BundleFusion and DVO SLAM are quoted from corresponding papers. The results indicate that our approach obtains comparable accuracy to the state-of-the-art methods. Figure 6 shows reconstruction results with the proposed approach, the first row for estimated camera trajectories compared with the ground truth and the second row for surface reconstruction models. From the diagram, it can be seen that the accuracy of camera trajectories produced by our approach is close to the ground truth. Reconstruction results show that our system achieves a good performance in indoor scene reconstruction. Besides, we also carry out experiments on four large scene sequences (Living Room 1–2 and Office 1–2) of the Augmented ICL-NUIM dataset. The average trajectory length of those sequences is 36 meters. Table 5 reports the accuracy of camera trajectories (ATE RMSE in meters) compared with other 3D reconstruction systems. Note that the results of Kintinuous, Choi et al.’s method [12], BundleFusion and DVO SLAM are quoted from corresponding papers. The experiments with InfiniTAM-v3 on the four sequences all failed due to tracking lost in the 1418th, 1510th, 296th, and 2285th frames, respectively. The results show that the average accuracy of camera trajectories with our method on the Augmented ICL-NUIM dataset is higher than the state-of-the-art methods.

**Figure 7:**
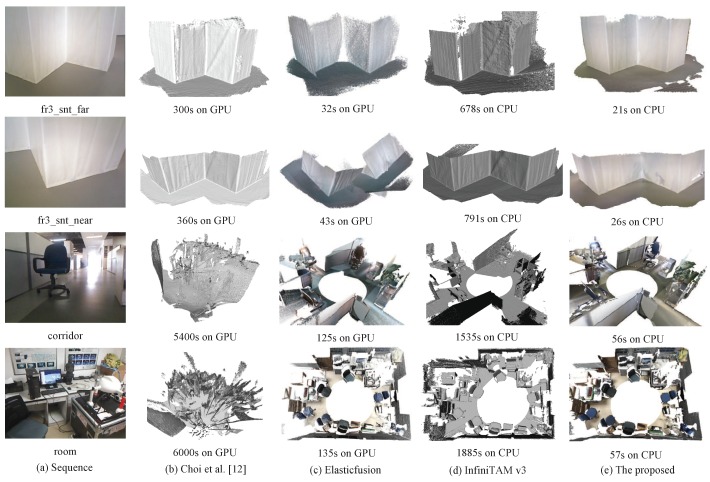
Comparison of the reconstruction results on real-world scenes. Fr3_snt_far and fr3_snt_near are manually scanned with the Asus Xtion sensor. Corridor and room are scanned through a robot equipped with Microsoft Kinect.

For the qualitatively evaluation, real-world scene experiments are carried out on the TUM RGB-D dataset and our dataset. Table 6 reports the accuracy of the estimated camera trajectories (RMSE in centimeters) and mean speeds (fps) of data fusion on the TUM RGB-D dataset. Note that the speeds of other methods are estimated from corresponding papers. The average accuracy of our approach is higher than others. Figure 7 shows a comparison of four indoor scenes’ reconstruction results on the TUM RGB-D dataset and our dataset. Fr3_snt_far (795 views) and fr3_snt_near (1055 views) are fully wrapped in a white plastic foil with little texture. They are manually scanned along a zig-zag structure. Their views used for integration are 651 and 783, respectively. Corridor (2047 views) and room (2215 views) are indoor scenes of our dataset and scanned through a robot equipped with Microsoft Kinect. The sequences have some redundancy since the movement of the robot is less flexible or more repeated. The views of corridor and room used for integration are 999 and 1329, respectively. The runtime marked in the figure is the total time of 3D reconstruction, i.e., camera tracking time and data fusion time. Choi et al.’s method [12] and ElasticFusion are run on an Nvidia GeForce GTX 750 Ti 2-GB GPU, while InfiniTAM v3 and our system are run on an Intel Core i7-4790 3.6-GHz CPU. The average speed of InfiniTAM-v3 is 1.25 Hz (fps) in our experiments. Compared to other methods, our approach produces better reconstruction results on the scenes containing textureless regions (fr3_snt_far, fr3_snt_near and corridor). All the results show that our system has a good performance and spends the shortest time even on the CPU.

## 6. Conclusions

We have presented a robust CPU-based approach to reconstruct indoor scenes scanned with a consumer RGB-D camera efficiently. The key idea is to estimate camera motion via points and edges and then integrate RGB-D images with an efficient data fusion strategy. Experimental results demonstrate the better performance of our proposed approach in terms of both robustness and efficiency. Our approach is applicable for indoor scene reconstruction on resource-constrained robots.

## Figures and Tables

**Table 1:** Accuracy of camera tracking (ATE RMSE in centimeters) compared to different tracking methods on the TUMRGB-D dataset (with an Intel Core i7-4790 CPU). Bold shows the best results. X denotes uninitialized or the tracking lost.

Methods	Point		Edge		Point and Line	Point and Edge
	ORB-SLAM		Edge VO	Edge SLAM	PL-SLAM	Our Method
	Monocular	RGB-D	Monocular	Monocular	Monocular	RGB-D
fr1_xyz	**0.90**	1.07	16.51	1.31	1.21	0.91
fr2_desk	**0.88**	0.90	33.67	1.75	-	0.92
fr2_xyz	**0.30**	0.40	21.41	0.49	0.43	0.37
fr3_st_near	1.58	1.10	47.63	1.12	1.25	**0.91**
fr3_st_far	0.77	1.06	121.00	**0.65**	0.89	1.02
fr3_snt_near	X	X	101.03	8.29	-	**2.11**
fr3_snt_far	X	6.71	41.76	6.71	-	**1.91**

**Table 2:** Mean computing speed of camera tracking on the TUM RGB-D dataset (with an Intel Core i7-4790 CPU). Bold shows the best results.

Operation	Point	Point and Line	Point and Edge
	ORB-SLAM	PL-SLAM	Our Method
Features Extraction (ms)	**10.76**	31.32	Point: **10.76**
			Edge: **21.08**
Initial Pose Estimation (ms)	7.16	7.16	**2.76**
Track Local Map (ms)	**3.18**	12.58	**3.18**
Total (fps)	50 Hz	20 Hz	**31–58 Hz**

**Table 3:** The accuracy of camera tracking (ATE RMSE in centimeters) via points and edges on the ICL-NUIM [38] and Augmented ICL-NUIM [12] datasets. Bold shows the best results. X denotes uninitialized or the tracking lost.

Methods		Point	Edge	Point and Edge
		ORB-SLAM [19]	Edge VO [25]	Our Method
		RGB-D	RGB-D	RGB-D
ICL-NUIM Living room	kt0	X	39.6	**0.55**
	kt1	0.77	27.7	**0.69**
	kt2	1.29	64.8	**1.22**
	kt3	**0.89**	114	0.94
ICL-NUIM Office	kt0	**3.26**	X	3.48
	kt1	X	92.9	**2.49**
	kt2	**1.88**	44.5	1.96
	kt3	1.36	27.3	**1.25**
Augmented ICL-NUIM	Living Room 1	3.71	X	**3.67**
	Living Room 2	1.09	112	**1.01**
	Office 1	X	176	**6.77**
	Office 2	**3.08**	178	3.13

**Table 4:** Accuracy of the estimated camera trajectories (ATE RMSE in centimeters) and surface reconstruction (median distance in centimeters) on the ICL-NUIM living room sequences [38]. Bold shows the best results.

Methods		Camera Trajectories (RMSE)					Surface Reconstruction (Median Distance)				
		kt0	kt1	kt2	kt3	Average	kt0	kt1	kt2	kt3	Average
GPU	Kintinuous [6]	7.2	0.5	1.0	35.5	11.05	1.1	0.8	0.9	15.0	4.45
	Choi et al. [12]	1.4	7.0	1.0	3.0	1.53	1.0	1.4	1.0	1.9	1.33
	ElasticFusion [9]	0.9	0.9	1.4	10.6	3.45	0.7	0.7	0.8	2.8	1.25
	InfiniTAMv3 [10]	0.9	2.9	0.9	4.1	2.20	1.3	1.1	**0.1**	1.4	0.98
	BundleFusion [14]	0.6	**0.4**	**0.6**	1.1	**0.68**	**0.5**	**0.6**	0.7	0.8	**0.65**
CPU	DVOSLAM [8]	10.4	2.9	19.1	15.2	11.90	3.2	6.1	11.9	5.3	6.63
	Our method	**0.5**	0.7	1.2	**0.9**	0.80	**0.5**	0.7	1.0	**0.7**	0.73

**Table 5:** Accuracy of the estimated camera trajectories (ATE RMSE in meters) on the Augmented ICL-NUIM dataset [12]. X denotes the tracking lost. Bold shows the best results.

Methods		Living Room 1	Living Room 2	Office 1	Office 2	Average
GPU	Kintinuous [6]	0.27	0.28	0.19	0.26	0.250
	Choi et al. [12]	0.10	0.13	0.13	0.09	0.113
	ElasticFusion [9]	0.62	0.37	0.13	0.13	0.313
	InfiniTAM v3 [10]	X	X	X	X	X
	BundleFusion [14]	**0.01**	**0.01**	0.15	**0.01**	0.045
CPU	DVO SLAM [8]	1.02	0.14	0.11	0.11	0.345
	Our method	0.04	**0.01**	**0.07**	0.03	**0.038**

**Table 6:** Accuracy of the estimated camera trajectories (ATE RMSE in centimeters) and mean speed (fps) of data fusion on the TUM RGB-D dataset [35]. Bold shows the best results.

Methods	Camera Trajectories (RMSE)					Mean Speed (fps)	
	fr1_desk	fr2_xyz	fr3_office	fr3_nst	Average	GPU	CPU
Kintinuous [6]	3.7	2.9	3.0	3.1	3.18	15 Hz	-
Choi et al. [12]	39.6	29.4	8.1	-	25.7	offline	-
ElasticFusion [9]	2.0	1.1	1.7	1.6	1.60	32 Hz	-
InfiniTAM v3 [29]	1.8	2.1	2.2	2.0	2.03	**910 Hz**	-
BundleFusion [14]	**1.6**	1.1	2.2	**1.2**	1.53	36 Hz	-
DVO SLAM [8]	2.1	1.8	3.5	1.8	2.30	-	30 Hz
Our method	**1.6**	**0.4**	**1.0**	1.9	**0.98**	-	**81 Hz**
